# Microglial Lyzl4 Facilitates β‐Amyloid Clearance in Alzheimer's Disease

**DOI:** 10.1002/advs.202412184

**Published:** 2024-11-18

**Authors:** Jie Pan, Jie Zhong, Ji Geng, Jane Oberhauser, Shihua Shi, Jun Wan

**Affiliations:** ^1^ Department of Pathology Stanford University School of Medicine Palo Alto CA 94305 USA; ^2^ Shenzhen Key Laboratory for Neuronal Structural Biology Biomedical Research Institute Shenzhen Peking University – The Hong Kong University of Science and Technology Medical Center Shenzhen Guangdong Province 518036 China; ^3^ Department of Systems Biology School of Life Sciences Southern University of Science and Technology Shenzhen Guangdong Province 518055 China; ^4^ Neuroscience Graduate Program University of California San Francisco San Francisco CA 94143 USA; ^5^ Friedrich Miescher Institute for Biomedical Research (FMI) Basel 4056 Switzerland; ^6^ Department of Neuroscience School of Life Sciences Southern University of Science and Technology Shenzhen Guangdong Province 518055 China

**Keywords:** Alzheimer's disease (AD), Lyzl4, microglia, phagocytosis, RNA‐seq

## Abstract

Alzheimer's Disease (AD) is a neurodegenerative condition characterized by the accumulation and deposition of amyloid‐β (Aβ) aggregates in the brain. Despite a wealth of research on the toxicity of Aβ and its role in synaptic damage, the mechanisms facilitating Aβ clearance are not yet fully understood. However, microglia, the primary immune cells of the central nervous system, are known to maintain homeostasis through the phagocytic clearance of protein aggregates and cellular debris. In this study, RNA sequencing analysis and live cell functional screens are employed to uncover microglial genetic modifiers related to AD. *Lyzl4* is identified, which encodes a c‐type lysozyme‐like enzyme primarily localized to microglial lysosomes, as a gene significantly upregulated in AD microglia with aging and propose that *Lyzl4* upregulation acts as a positive regulator of Aβ clearance. Furthermore, it is found that *Lyzl4* overexpression boosts Aβ clearance both in vitro and in vivo, underscoring its potential for mitigating Aβ burden. These novel insights position *Lyzl4* as a promising therapeutic target for Alzheimer's disease, paving the way for further exploration into potential AD treatments.

## Graphical abstract

Alzheimer's Disease (AD) features amyloid‐β (Aβ) buildup in the brain, with microglia playing a crucial role in clearing such aggregates. This study highlights Lyzl4, a gene upregulated in AD microglia, as crucial for enhancing Aβ clearance. Lyzl4 overexpression significantly improved Aβ removal in both cell and animal models, suggesting its potential as a therapeutic target to reduce Aβ burden in AD.

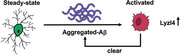

## 1 Introduction

Alzheimer's disease (AD) represents a major healthcare challenge of the 21st century and the leading cause of dementia^[^ ^1^ ^]^ As a well‐known hallmark of AD pathology, amyloid‐β (Aβ) aggregation has drawn extensive attention and has been intensively studied since its discovery by Alois Alzheimer.^[^ ^2^ ^]^ Aβ peptides are produced via the proteolytic cleavage of amyloid precursor protein (APP). Improper cleavage of APP is believed to initiate or even drive AD progression, at least in familial cases.^[^ ^3^ ^]^ In healthy individuals, a balance exists between the production and clearance of Aβ. In Alzheimer's disease, this balance is disrupted, leading to the accumulation of Aβ plaques in the brain. Extracellular Aβ has been shown to cause significant toxicity, resulting in synaptic damage, increased reactive oxidative stress, and leading to microglial infiltration around plaque areas^[^ ^4^ ^]^

Microglia are the resident macrophages of the central nervous system (CNS), where they perform a range of different functions.^[^ ^5^ ^]^ To maintain CNS homeostasis, microglia continually remove toxic protein plaques, cell debris, pathogens, and damaged or dying cells from the tissue they inhabit.^[^ ^6^ ^]^ While once considered secondary events to disease progression, the cell‐autonomous role of microglial activation in AD has become increasingly clear.^[^ ^7^ ^]^ Genetic analyses have revealed that many genes associated with increased risk for AD and other neurodegenerative disorders are particularly expressed by microglia in the CNS. As a result of these findings, interest in these macrophages in the context of neurodegeneration has grown significantly.^[^ ^8^ ^]^ In AD, microglia bind to Aβ via scavenger receptors, including the class A scavenger receptor (SR‐A), class B scavenger receptor (CD36), and the receptor for advanced glycation end products (RAGE).^[^ ^4^, ^9^, ^10^, ^11^, ^12^ ^]^ Following receptor binding, microglia recognize and endocytose Aβ oligomers, which are eliminated by endosomal–lysosomal degradation.^[^ ^13^, ^14^, ^15^ ^]^ The endosomal‐lysosomal system is composed of dynamically communicating vesicles that sort and traffic internalized extracellular materials (nutrients, trophic factors, etc.) to sites throughout the cell. Upon binding to Aβ, microglia phagocytose, or engulf, Aβ plaques, effectively removing them from the brain tissue.

Microglial phagocytosis is facilitated by various receptors and signaling pathways. For example, the triggering receptor expressed on myeloid cells 2 (TREM2) is a receptor expressed on microglia that recognizes Aβ and stimulates its phagocytosis.^[^ ^11^ ^]^ Other receptors, such as CD33, also influence microglial response and Aβ clearance.^[^ ^16^ ^]^ Microglia can also release enzymes, such as neprilysin, insulin‐degrading enzyme (IDE), and matrix metalloproteinases (MMPs), which degrade Aβ peptides and facilitate their clearance.^[^ ^17^, ^18^ ^]^ Additionally, microglia release cytokines, chemokines, and other immune molecules that modulate the inflammatory response in the brain and contribute to the recruitment of other immune cells to the site of Aβ deposition.^[^ ^19^ ^]^

Lysozymes, traditionally recognized for their antimicrobial properties, have been increasingly studied in the context of neurodegenerative diseases, including AD. Previous studies have demonstrated that lysozymes exert protective effects against Aβ pathology in *Drosophila melanogaster*, AD mouse models, and human AD cases.^[^ ^20^, ^21^, ^22^ ^]^ Evidence suggests that lysozymes can bind to Aβ, potentially influencing its aggregation and clearance and thereby impacting one of the primary pathological features of AD. While lysozyme‐like proteins share structural and enzymatic similarities with lysozymes, the precise roles of these proteins within the CNS, particularly in the context of chronic neurodegeneration, remain unresolved. Understanding how lysozyme‐like proteins interact with AD pathology could provide new insights into mechanisms of disease progression and potential therapeutic targets.

Here, we report for the first time that Lyzl4 is upregulated in microglia from aged APP/PS1 mice. Lyzl4 is a member of the c‐type lysozyme‐like family strongly expressed in the testis and epididymis and expressed at relatively low levels in the brain.^[^ ^23^ ^]^ Previous studies have primarily focused on the role of Lyzl4 in the reproductive system and its role in sperm‐egg binding, hyaluronan interaction, and free radical scavenging.^[^ ^24^ ^]^ However, its CNS‐specific expression outside the reproductive system makes *Lyzl4* a gene of particular interest. In our study, we find that microglial overexpression of Lyzl4 results in enhanced Aβ clearance both in vitro and in vivo, indicating the potential role of microglial Lyzl4 in attenuating Aβ burden. These findings hint that Lyzl4 might present a promising therapeutic target in AD.

## 2 Results

### 2.1 Elevated Levels of Lyzl4 are Present in Microglia from Aging APP‐PS1 Mice

**Figure 1:**
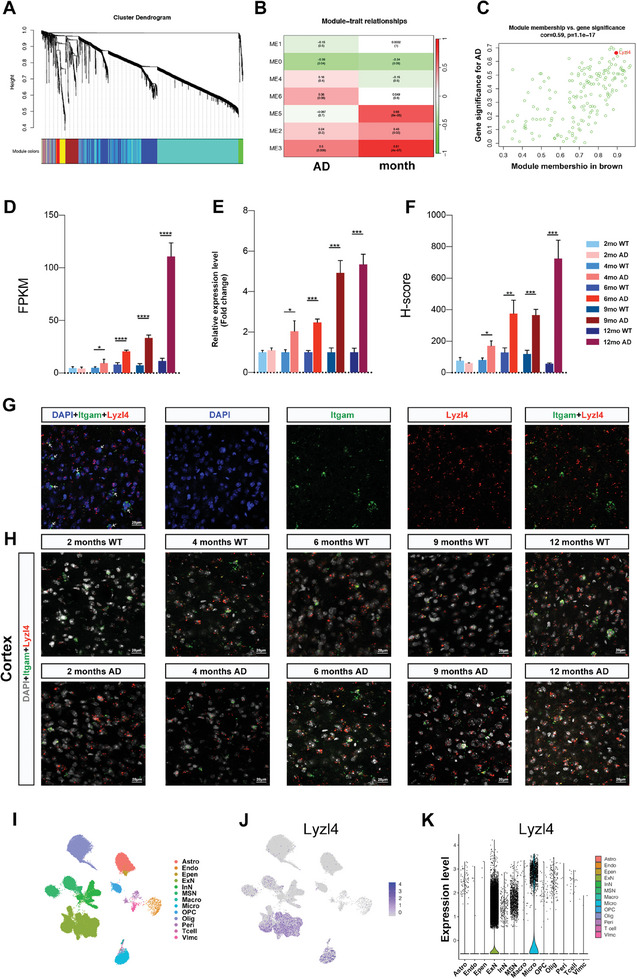
Upregulation of Lyzl4 in microglia from APP/PS1 mice. A) Gene dendrogram obtained by average linkage hierarchical clustering, produced by WGCNA. Modules of co‐expressed genes are represented by the different colored bars below the dendrogram. Each module is assigned a unique color for easy identification. B) Heatmap of module‐trait relationships illustrating the correlation between gene modules (identified in panel A) and clinical traits (AD and mouse age). Each cell contains a correlation coefficient and corresponding p‐value (in parentheses). The intensity of color filling each cell represents the strength of the correlation, with red indicating a positive correlation and green indicating a negative correlation. C) Scatterplot of gene significance gene significance for AD (y‐axis) versus module membership (x‐axis) in the most significant module (M3: brown module, see panel B). The gene Lyzl4, highlighted in red, is shown to have high module membership and high gene significance for AD. D) RNA‐seq analysis showing Lyzl4 gene expression in primary microglia from 2, 4, 6, 9, and 12‐month old mice. At each time point, differential expression analysis was performed on microglia from AD and WT mice using DEseq2. Data are shown as FPKM means ± SEM; ^****^ *p* < 0.0001, ^***^ *p* < 0.001, ^**^ *p* < 0.01, ^*^ *p* < 0.05; *n* = 3 animals. E) RT–qPCR analysis of Lyzl4 expression in acutely isolated primary microglia at five‐time points, normalized to a housekeeping gene (Gapdh). Data represent fold change relative to WT microglia. Unpaired t‑test; data are shown as means ± SEM; ^****^ *p* < 0.0001, ^***^ *p* < 0.001, ^**^ *p* < 0.01, ^*^ *p* < 0.05; *n* = 3 animals. F) H‐score quantification of Itgam^+^ microglia expressing detectable levels of Lyzl4 mRNAs at five‐time points. Unpaired t‑test; data are shown as means ± SEM; ^****^ *p* < 0.0001, ^***^ *p* < 0.001, ^**^ *p* < 0.01, ^*^ *p* < 0.05; *n* = 3 animals. G) Representative epifluorescence images with microglial marker Itgam (green), Lyzl4 (red), and nuclei (DAPI, blue) in cortex. White arrowheads indicate Itgam^+^ Lyzl4^+^ microglia. Scale bar: 20 µm. H) Representative in situ hybridization images for Lyzl4 illustrating colocalization of Lyzl4 with Itgam in the AD and WT mouse cortex at 2, 4, 6, 9, and 12‐month age intervals. Scale bar: 20 µm. I) UMAP plot illustrating the clustering of different cell types from the aging mouse brain^[^ ^30^ ^]^. Cell types are color‐coded. J) UMAP plot displaying the expression level of Lyzl4 across different cell clusters. Color intensity indicates the expression level. K) Violin plot illustrating the distribution of Lyzl4 expression across various cell types. Lyzl4 expression is highest in ExN and microglia cell types.

To identify key genes potentially involved in Alzheimer's disease (AD), we first collected primary microglia from APP/PS1 (AD) mice and wild‐type (WT) mice at various ages (2, 4, 6, 9, and 12 months) and performed RNA sequencing, as described in previous studies.^[^ ^25^ ^]^ Using Weighted Gene Co‐Expression Network Analysis (WGCNA),^[^ ^26^ ^]^ we constructed a gene co‐expression network to identify modules of highly correlated genes. Hierarchical clustering and module assignment revealed seven distinct gene modules (**Figure** **1**A). We then correlated these modules with AD status and mouse age, identifying significant module‐trait relationships. The ME3 module exhibited a strong positive correlation with both AD progression and age (Figure 1B). To further narrow down the key genes within the ME3 module, we examined the relationship between module membership and gene significance for AD (Figure 1C). In modules related to a trait of interest, genes with high module membership often also have high gene significance. This analysis highlighted Lyzl4 as having both high module membership and high gene significance for AD. Based on these comprehensive analyses, we selected Lyzl4 for subsequent experimental validation. Its significant correlation with disease traits in our dataset suggested that Lyzl4 may play a crucial role in the pathogenesis of AD.

To validate the RNA sequencing results, we conducted qPCR using samples from a new batch of mice at five different time points. Data were expressed as 2^−ΔΔCt^ using the Gapdh transcript as an internal reference standard. qPCR yielded results consistent with RNA‐seq (Figure 1D,E). In addition, we synthesized a Lyzl4 probe and conducted RNAscope on microglia in the hippocampus, cortex, and cerebellum of WT and AD mice. We determined the total number of dual‐positive cells for Itgam^+^ microglia also expressing Lyzl4. Additionally, we quantified the target‐probe dots per cell and the total number of signal dots from 100 cells in the cortex, 50 cells in the hippocampus, and 50 cells in the cerebellum (Figure 1G,H). Our in situ hybridization results concurred with the FPKM (Fragments Per Kilobase per Million mapped fragments) values obtained from RNA‐seq and qPCR, which indicated that Lyzl4 was significantly upregulated with age in AD microglia (Figure 1F).

We further validated changes in Lyzl4 expression in another AD mouse model. APP^SAA^ is a new knock‐in mouse model that exhibits earlier disease‐relevant biology and the progressive accumulation of parenchymal amyloid plaques and vascular amyloid deposits of AD.^[^ ^27^ ^]^ In the APP^SAA^KI/KI mouse model, Lyzl4 expression was markedly elevated at 8 months of age compared to WT controls (Figure S1A, Supporting Information). In rTg4510 mice that model AD tau pathology,^[^ ^28^ ^]^ Lyzl4 expression levels showed a significant increase at 4, 6, and 8 months compared to their WT counterparts (Figure S1B, Supporting Information), suggesting that Lyzl4 might be a genetic factor contributing to the progression of AD.

### 2.2 Lyzl4 is Located in the Lysosomes of Microglia and is Highly Conserved

**Figure 2:**
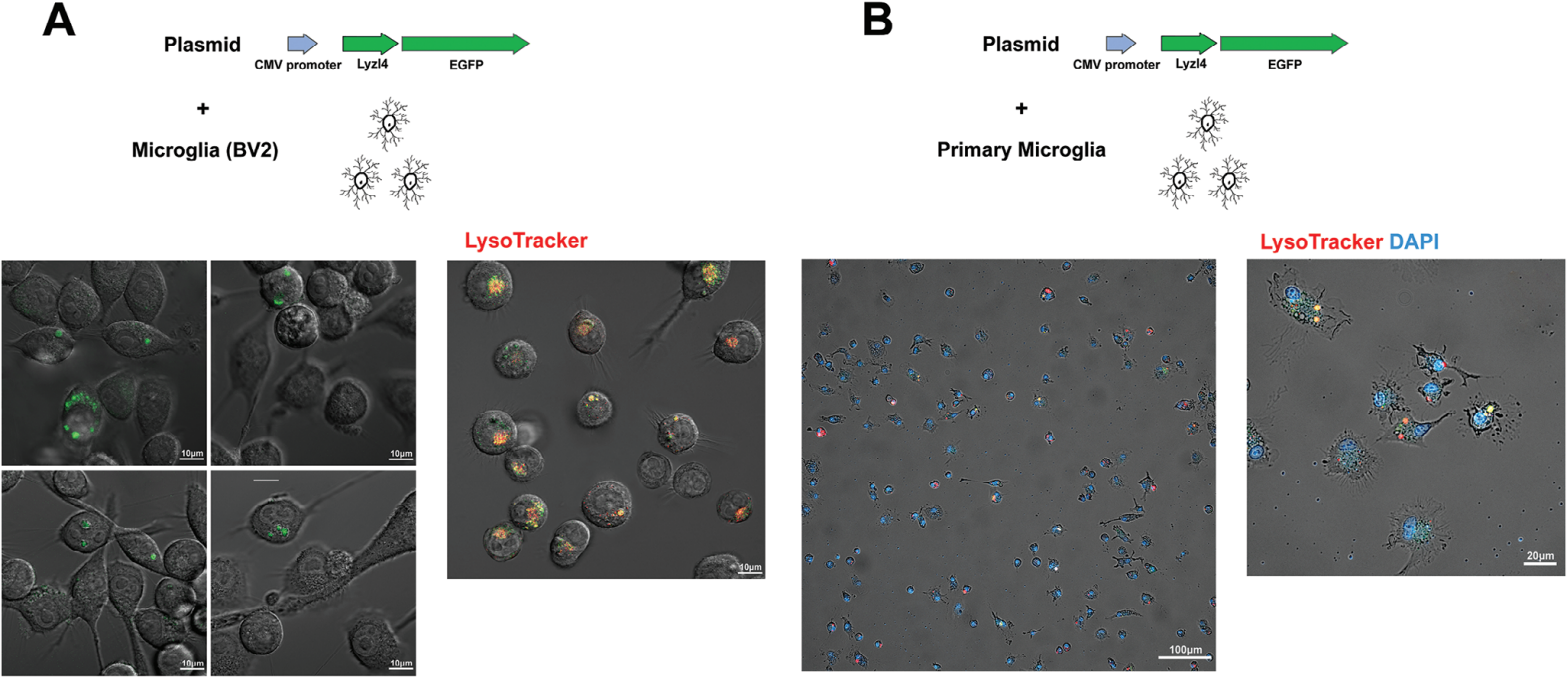
Lyzl4 localizes to the lysosomes of microglia. A) The BV2 microglial cell line was transfected with CMV‐Lyzl4‐EGFP plasmids. Representative epifluorescence images illustrate the co‐localization of lyzl4 (green) and lysosomes (red) in BV2 cells. B) Primary microglia transfected with CMV‐Lyzl4‐EGFP plasmids. Representative epifluorescence images illustrate the co‐localization of lyzl4 (green) and lysosomes (red) in primary microglia.

The c‐type lysozyme‐like family comprises four members: Lyzl1, Lyzl3, Lyzl4, and Lyzl6. Our results indicate that Lyzl4 is the only member of its family expressed in brain tissue, while all four family members are present in the reproductive system (Figure S1I, Supporting Information). Lyzl4 shows the highest levels of tissue‐specific expression in the reproductive system, followed by the brain (Figure S1H, Supporting Information). Comparative analysis of amino acid sequences among the c‐type lysozyme‐like proteins, as well as across different species, reveals that Lyzl4 is highly conserved, sharing ≈60% homology between species (Figure S1F,G, Supporting Information). Despite its classification as a lysozyme‐like protein, Lyzl4 lacks lysozyme activity, as demonstrated in previous research.^[^ ^23^, ^29^ ^]^ The catalytic domain region of Lyzl4 differs from that of other lysozyme proteins due to a 52nd amino acid mutation (D52G) and a 70–74th amino acid deletion. Additionally, the Allen Institute's single‐cell sequencing data^[^ ^30^ ^]^ indicates that Lyzl4 is primarily expressed in microglia and excitatory neurons within the mouse central nervous system (Figure 1I–K). In the absence of commercially available antibodies for the detection of Lyzl4 in rodents, we created CMV‐Lyzl4‐EGFP plasmids to observe Lyzl4 subcellular localization in the microglial cell line BV2 and in primary microglia. Live‐cell imaging revealed that Lyzl4 is predominantly localized within the lysosomal compartments of microglia (**Figure** **2**A,B).

### 2.3 Aβ Increases the Expression of Lyzl4 in Microglia

**Figure 3:**
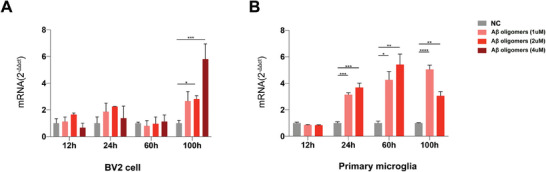
Upon treatment with Aβ oligomers, microglial Lyzl4 expression proves time course and dose‐dependent. A) RT–qPCR analysis of Lyzl4 expression in BV2 cells incubated with Aβ oligomers (1, 2, and 4 µm) or negative control. Cells were treated for 12, 24, 60, or 100 h. One‐way ANOVA followed by Dunnett post hoc test; data are shown as means ± SEM; ^****^ *p* < 0.0001, ^***^ *p* < 0.001, ^**^ *p* < 0.01, ^*^ *p* < 0.05; *n* = 3. B) RT–qPCR analysis of Lyzl4 expression in primary microglia incubated with Aβ oligomers (1 and 2 µm) or negative control. Cells were treated for 12, 24, 60, or 100 h. One‐way ANOVA followed by Dunnett post hoc test; data are shown as means ± SEM; ^****^ *p* < 0.0001, ^***^ *p* < 0.001, ^**^ *p* < 0.01, ^*^ *p* < 0.05; *n* = 3.

Aβ, a particularly neurotoxic protein aggregate that accumulates in the AD brain, has been considered a major inducer of neuroinflammation in AD and a cause of central nervous system disruption.^[^ ^31^ ^]^ To explore the potential relationship between Lyzl4 and Aβ in AD, we isolated and cultured both primary microglia and BV2 cells in vitro, then stimulated the cells with oligomeric Aβ_1‐42_ at concentrations of 1, 2, and 4 µm. According to our qPCR analysis, the exposure of BV2 cells to Aβ_1‐42_ oligomers at concentrations of 2 or 4 um for a duration of 100 h led to an increase in Lyzl4 expression (**Figure** **3**A). Similarly, treating primary microglial cells with Aβ_1‐42_ oligomers at a concentration of 1 or 2 um for a shorter period of 24 h also triggered a significant upregulation of Lyzl4 expression. Additionally, an increase in Lyzl4 expression was observed in primary microglia treated with 1 or 2 µm Aβ_1‐42_ oligomers at both 60 and 100 h time points (Figure 3B). Due to the cytotoxicity observed with 4 µM Aβ_1‐42_ oligomers in primary microglial cells, data for this concentration are not presented.

### 2.4 Overexpression of Lyzl4 in Microglia Promotes Aβ Clearance In Vitro

Microglia have been demonstrated to play a crucial role in the internalization and degradation of Aβ in AD.^[^ ^32^ ^]^ Moreover, our observations indicate that Aβ stimulation increases Lyzl4 expression, which we have shown is mainly localized to microglial lysosomes. Hence, we hypothesize that Lyzl4 is implicated in the lysosomal clearance of Aβ by microglia. To test this hypothesis, we initially examined the effect of Lyzl4 on microglial Aβ degradation in vitro.

In order to minimize non‐specific background interference in our phagocytosis assays, we employed pHrodo, a pH‐sensitive reagent capable of binding to amino acids or peptides. Two varieties of phagocytosis materials were manufactured: pHrodo‐Polybead‐Amino Microspheres, a commonly used material for assessing phagocytosis, and pHrodo‐Aβ oligomers. When excited by a laser in acidic environments, both phagocytosis‐asessing materials emit fluorescence; in contrast, no signal is emitted in neutral or alkaline environments. As a result, the 488 nm laser will not excite extracellular phagocytosis reagents. Fluorescence will only be emitted when pHrodo‐Polybead‐Amino Microspheres or pHrodo‐Aβ oligomers are phagocytosed and reach the highly acidic environment of the microglial lysosomes.

To overcome the low microglial transfection efficiency associated with conventional methods, we constructed a lentiviral vector containing an iRES‐tomato red fluorescent protein for the infection of microglia (Figure S2A, Supporting Information). Accordingly, both transfection efficiency and overexpression efficiency were high, with a viral titer of 60 MOI (Figure S2B, Supporting Information). The resulting Lyzl4‐overexpressing microglia were co‐cultured with pHrodo‐Polybead‐Amino Microspheres and pHrodo‐Aβ oligomers. After treatment with the actin‐polymerization inhibitor Cytochalasin D, functional phagocytosis was completely lost (Figure S2C, Supporting Information), whereas phagocytic function was observed as expected in the absence of Cytochalasin D treatment (Figure S2D,E, Supporting Information). These results indicate that the phagocytosis materials were produced successfully.

**Figure 4:**
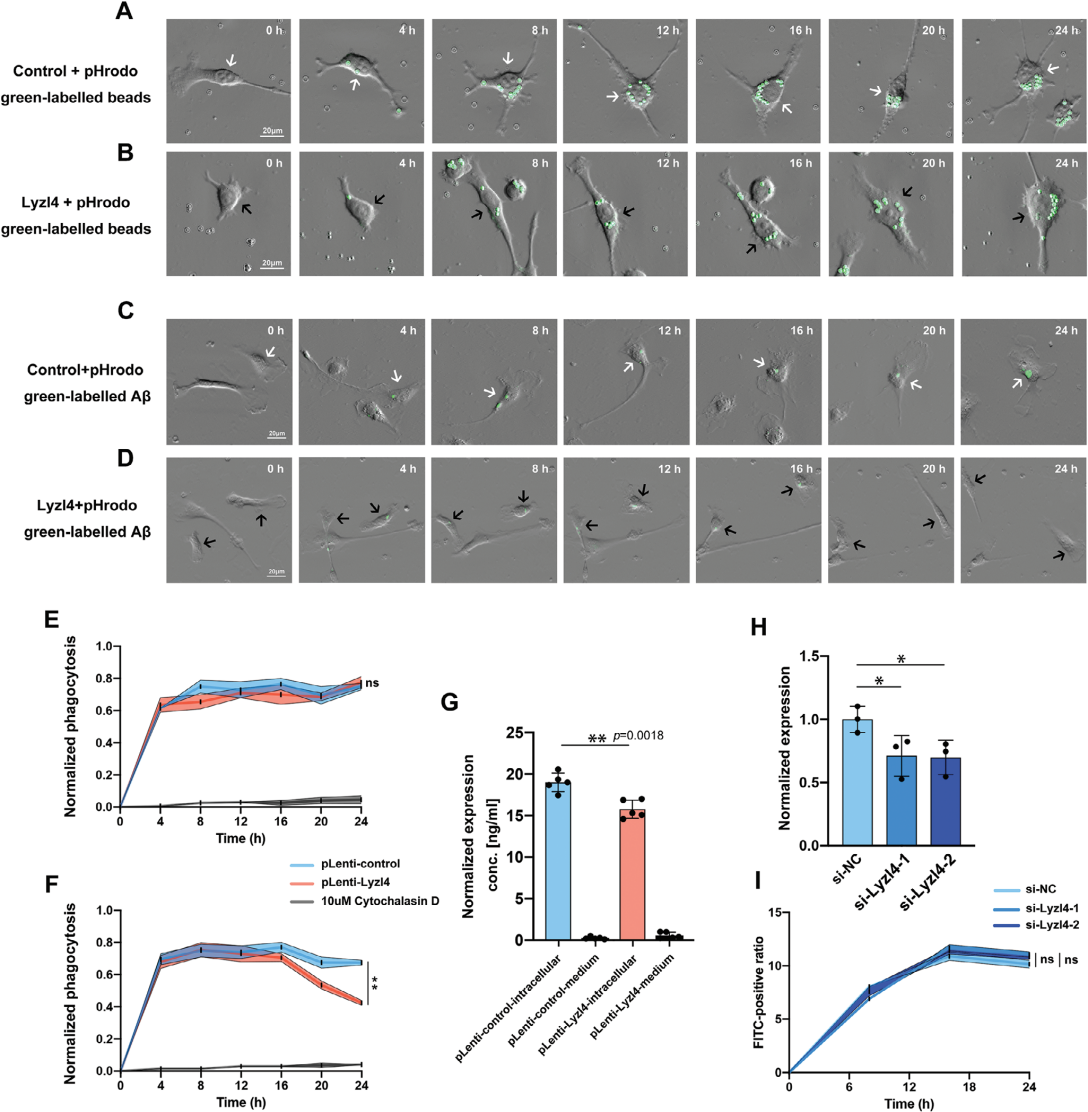
Lyzl4 overexpression in primary microglia enhances the clearance of Aβ without altering phagocytosis. A,B) Representative images of control (A) and Lyzl4‐overexpressing (B) primary microglia that have taken up pH‐sensitive green‐labeled fluorescent beads. Images were taken every 4 h for a total of 24 h (0, 4, 8, 12, 16, 20, and 24 h). The white arrow indicates a single control microglia and the beads it takes up over time. The black arrow indicates a single Lyzl4‐overexpressing microglia and the beads it takes up over time. Scale bar: 20 µm. C,D) Representative images of control (C) and Lyzl4‐overexpressing (D) primary microglia that have taken up pH‐sensitive green‐labeled fluorescent Aβ. Images were taken every 4 h for a total of 24 h (0, 4, 8, 12, 16, 20, and 24 h). The white arrow indicates a single control microglia and the green‐labeled fluorescent Aβ it takes up over time. The black arrow indicates a single Lyzl4‐overexpressing microglia and the green‐labeled fluorescent Aβ it takes up over time. Scale bar: 20 µm. E) Normalized phagocytosis levels (green fluorescent microglia/ total microglia) monitored over 24 h. Primary microglia were co‐incubated with pH‐sensitive green‐labeled fluorescent beads. Microglia were treated with pLenti‐Lyzl4, pLenti‐control, or 10 µm cytochalasin D. We observed no significant differences in the phagocytosis of beads between the pLenti‐Lyzl4 and pLenti‐control group. Unpaired t‑test; data are shown as means ± SEM; ^****^ *p* < 0.0001, ^***^ *p* < 0.001, ^**^ *p* < 0.01, ^*^ *p* < 0.05; *n* = 5. F) Normalized phagocytosis levels (green fluorescent microglia/ total microglia) monitored over 24 h. Primary microglia were co‐incubated with pH‐sensitive green‐labeled fluorescent Aβ. Microglia were treated with pLenti‐Lyzl4, pLenti‐control, or 10 µm cytochalasin D. Lyzl4‐overexpressing cells displayed enhanced Aβ clearance after 24 h, but no significant difference was observed for the phagocytosis of Aβ from 4 to 16 h. Unpaired t‑test; data are shown as means ± SEM; ^****^ *p* < 0.0001, ^***^ *p* < 0.001, ^**^ *p* < 0.01, ^*^ *p* < 0.05; *n* = 5. G) Normalized Aβ levels were measured using ELISA in pLenti‐control‐intracellular, pLenti‐control‐medium, pLenti‐Lyzl4‐ intracellular, and pLenti‐Lyzl4‐medium after 24 h. Unpaired t‑test; data are shown as means ± SEM; ^****^ *p* < 0.0001, ^***^ *p* < 0.001, ^**^ *p* < 0.01, ^*^ *p* < 0.05; *n* = 5. H) Quantification of Lyzl4 mRNA levels in microglial cells transfected with control siRNA (si‐NC) and two different siRNAs targeting Lyzl4 (si‐Lyzl4‐1 and si‐Lyzl4‐2). Expression levels are normalized to the control group. One‐way ANOVA followed by Dunnett post hoc test; data are shown as means ± SEM; ^*^ *p* < 0.05. I) Time‐course analysis of Aβ‐positive microglia following Lyzl4 knockdown. Microglial cells transfected with si‐NC, si‐Lyzl4‐1, and si‐Lyzl4‐2 were treated with Aβ_1‐42_ oligomers and the proportion of FITC‐positive microglia was measured at 0, 8, 16, and 24 h post‐treatment. Data are presented as mean ± SEM (*n* = 3). No significant differences (ns) were observed between the groups at any time point.

To investigate whether Lyzl4 influences the phagocytic function of microglia, we used time‐lapse live cell imaging to observe and record the dynamic process of Lyzl4‐overexpressing microglia phagocytosing pHrodo‐Polybead‐Amino Microspheres and pHrodo‐Aβ_‐_oligomers (Videos [Link], [Link], [Link], [Link], [Link], Supporting Information). We recorded the percentage of GFP^+^ cells co‐cultured with the two phagocytic materials at various time points (0, 4, 8, 12, 16, 20, and 24 h) in normal control and Lyzl4‐overexpressing groups (**Figure** **4**A–D). The percentage of GFP^+^ cells did not differ significantly between the control and Lyzl4‐overexpressing groups co‐cultured with pHrodo‐Polybead‐Amino Microspheres (Figure 4E). This suggests that Lyzl4 does not influence overall microglial phagocytosis. However, in microglia co‐cultured with pHrodo‐Aβ oligomers, the proportion of GFP^+^ cells in the Lyzl4 overexpression group began to decrease at 16 h. At 24 h, Lyzl4‐overexpressing microglia displayed significantly fewer GFP+ cells compared to controls. This result suggests that Lyzl4 might be involved in the microglial clearance of pHrodo‐Aβ oligomers. In contrast, the percentage of GFP^+^ cells in the phagocytically‐impaired Cytochalasin D group remained close to zero across all time points (Figure 4F).

Moreover, ELISA was used to determine the intracellular Aβ concentrations in each experimental group. Intracellular Aβ concentrations were lower in the Lyzl4‐overexpressing microglia than in the control group, indicating that Lyzl4‐overexpressing microglia have a greater capacity for the clearance of pHrodo‐Aβ_‐_oligomers. In addition, Aβ levels in the supernatants of both groups were exceedingly low, indicating that extracellular Aβ was ingested by the microglia and its rate of uptake was unaffected by Lyzl4 overexpression (Figure 4G).

To further investigate the role of Lyzl4 on microglial responses to Aβ, we performed siRNA‐mediated knockdown of Lyzl4 expression in primary microglia (Figure 4H). Following Lyzl4 knockdown, microglia were treated with 1 µm Aβ_1‐42_ oligomers. Immunofluorescence staining was conducted to quantify the number of Aβ‐positive microglia at 0, 8, 16, and 24 h post‐treatment. In both the knockdown and control groups, the proportion of Aβ‐positive microglia increased steadily during the first 18 h, followed by a decrease at 24 h, indicating Aβ degradation. However, no significant differences were observed between the Lyzl4 knockdown group and the control group (Figure 4I). This result suggests that the inhibition of Lyzl4 alone is insufficient to inhibit microglial clearance. There might be other compensatory mechanisms/factors at play.

### 2.5 Microglial Lyzl4 Attenuates Aβ Burden In Vivo and is Potentially Associated with Reduced Inflammation and Micropinocytosis

**Figure 5:**
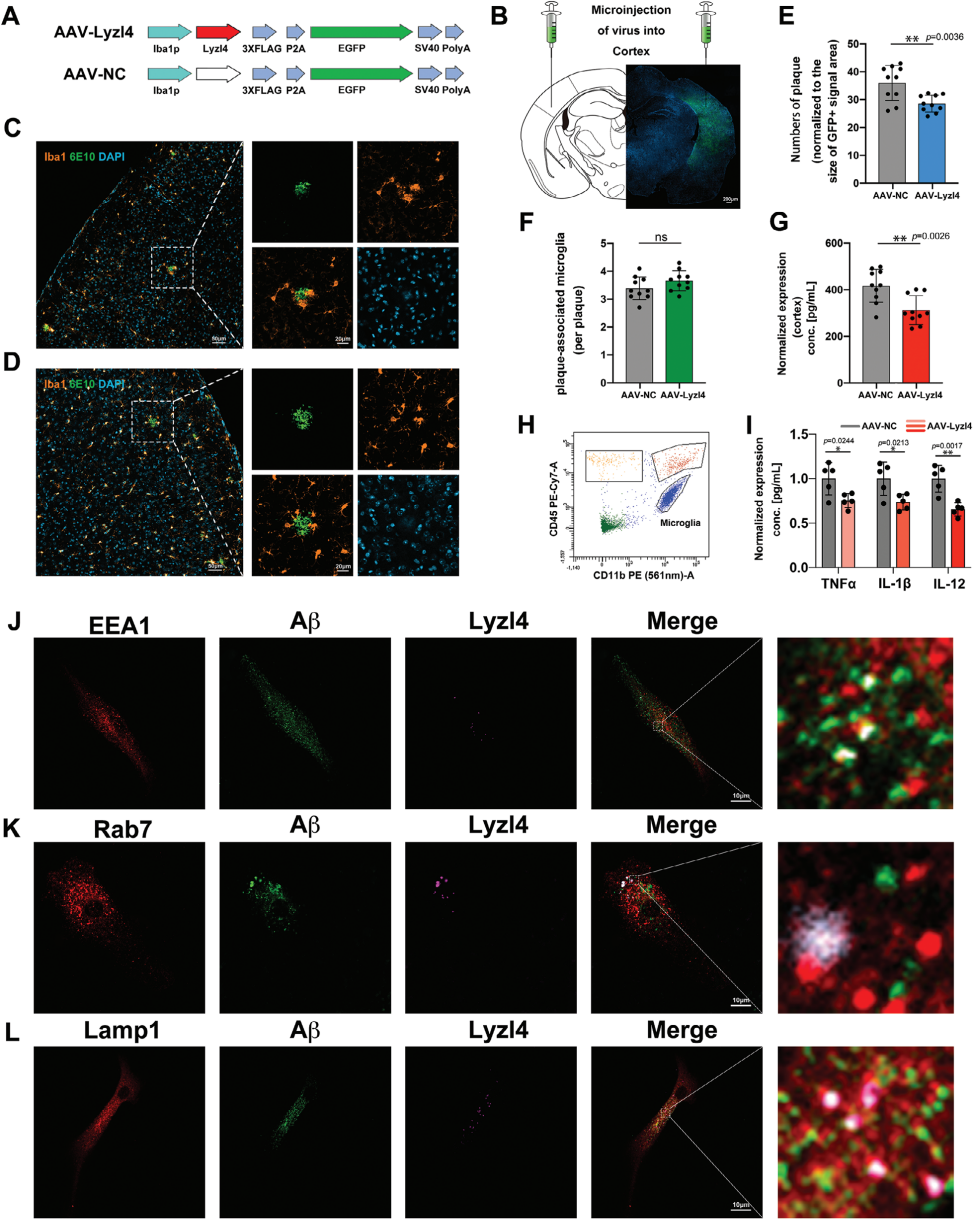
Lyzl4 facilitates microglial clearance of Aβ plaques in vivo and is potentially associated with macropinocytosis. A,B) Labeled AAV‐NC or AAV‐Lyzl4 was stereotactically injected bilaterally into the cortex of 5‐month‐old APP/PS1 mice (*n* = 5). One month later, frozen brain sections were collected prepared for immunohistochemistry. C,D) Representative epifluorescent images of microglial marker Iba1 (orange), Aβ (green), and nuclei (DAPI, blue) in an AAV‐Lyzl4 hemisphere (C) and an AAV‐NC hemisphere (D). Scale bar: 50 µm. E) Number of plaques in ROI from AAV‐NC versus AAV‐Lyzl4 mice, assessed 1 month after injection. Unpaired t‑test; data are shown as means ± SEM; ^**^ *p* < 0.01; *n* = 10 (2 slices per mouse, 5 mice). F) Numbers of plaque‐associated microglia (per plaque) in ROI from AAV‐NC versus AAV‐Lyzl4 mice, assessed 1 month after injection. Unpaired t‑test; data are shown as means ± SEM; *n* = 10 (2 slices per mouse, 5 mice). G) Normalized Aβ levels were measured using ELISA in cortical tissue from AAV‐NC versus AAV‐Lyzl4 mice. Data are shown as means ± SEM; ^**^ *p* < 0.01; *n* = 10 (2 slices per mouse, 5 mice). H) FACS plot exemplifying the gating strategy used to isolate microglial cells from brain regions treated with AAV‐Lyzl4 and AAV‐NC. The gating strategy identified microglia as CD11b^+^ CD45^low‐to‐medium^ cells. I) Normalized expression levels of the TNF‐α, IL‐1β, and IL‐12 in isolated microglia measured using the Milliplex biomarker assay. Unpaired t‐test; data are shown as means ± SEM; ^*^ *p* < 0.05, ^**^ *p* < 0.01. J–L) Representative epifluorescent images of Lyzl4 (purple), Aβ (green), and early endosome marker EEA1 (G), late endosome maker Rab7 (H), and lysosome marker Lamp1 (I) (red) in microglia. Scale bar: 10 µm.

Since Lyzl4 overexpression increased microglial‐mediated degradation of Aβ, we next investigated the consequences of this potentially beneficial effect in vivo. We engineered an adeno‐associated viral (AAV) vector containing the Lyzl4 gene under the control of a microglia‐specific promoter (**Figure** **5**A). To improve the efficacy of AAV diffusion in the cortex, we chose the AVV9 serotype. We delivered AAV‐Lyzl4 and AAV‐NC (control) bilaterally to the brains of 5‐month‐old APP/PS1 mice (*n* = 5) using stereotactic injection (Figure 5B). Then, we compared regions of interest (ROI; GFP+) between AAV‐Lyzl4 and AAV‐NC hemispheres. Immunohistochemistry demonstrated that Aβ plaques were substantially diminished and smaller (Figure 5C–E) in AAV‐Lyzl4 tissue, while the number of microglia per plaque remained unchanged (Figure 5F). This result indicates that Lyzl4 alleviates Aβ burden not by increasing the number of microglia surrounding Aβ plaques, but by enhancing microglial clearance capacity. Correspondingly, ELISA analysis of Aβ levels in the cortical hemisphere revealed that AAV‐Lyzl4 substantially decreased Aβ levels compared to AAV‐NC, confirming the role of Lyzl4 in promoting Aβ clearance (Figure 5G).

In addition, we collected microglia from the AAV‐Lyzl4 and the AAV‐NC treated sides of the brain using fluorescence‐activated cell sorting (FACS) with an appropriate gating strategy of CD11b^+^ CD45^low‐to‐medium^ (Figure 5H). The levels of proinflammatory cytokines, such as tumor necrosis factor (TNF)‐α, interleukin (IL)‐1β, and IL‐12 in the isolated microglial cells were measured using the Milliplex biomarker assay. We observed decreased levels of TNF‐α, IL‐1β, and IL‐12 in microglia from the AAV‐Lyzl4‐treated side, suggesting that overexpression of Lyzl4 in vivo contributes to reduced inflammation (Figure 5I). These findings indicate that Lyzl4 may play a protective role in regulating microglial inflammatory responses.

Finally, Aβ is taken up by endosomes, which ultimately fuse with autophagosomes/lysosomes for degradation.^[^ ^33^ ^]^ A failure to degrade aggregated Aβ_1–42_ in late endosomes or secondary lysosomes is a mechanism known to contribute to the intracellular accumulation of Aβ in AD.^[^ ^34^ ^]^ In order to investigate the potential mechanisms by which Lyzl4 promotes the degradation of Aβ in microglia, we infected primary microglia cultured on coverslips with pLVX‐IRES‐tdTomato lentivirus and treated them with a 2 µm Aβ oligomer solution. After 30 min of incubation, the cells were fixed and imaged to examine the co‐localization of Aβ, Lyzl4, and immunofluorescent markers of early endosomes (EEA1), late endosomes (Rab7), and lysosomes (Lamp1). Our results indicate that Aβ and Lyzl4 mainly co‐localize with Rab7 and Lamp1, but not EEA1 (Figure 5J–L), suggesting that Lzyl4 may promote Aβ clearance by modulating the trafficking process of Aβ to late endosomes and lysosomes following internalization.

## 3 Discussion

In this study, we combine transcriptomic analysis, genetic analysis, in vitro time‐lapse imaging, and stereotactic injection to demonstrate that stimulation with Aβ induces the transition of microglia from a steady state to an activated phenotype featuring the upregulation of Lyzl4. Upregulated Lyzl4 expression, in turn, enhances the microglial clearance of Aβ both in vitro and in vivo. This process represents a regulatory feedback loop executed by the CNS in an effort to adapt to environmental changes. The ability of microglia to effectively clear Aβ may represent a determining factor in AD pathogenesis.^[^ ^35^, ^36^ ^]^ Our findings suggest that modulating microglial Lyzl4 expression may prove beneficial for the treatment of AD and provide an avenue for further research on the molecular mechanisms underlying microglial involvement in Aβ clearance.

Aβ exists in both soluble and fibrillar forms in the brain. Microglia interact differently with these two forms of Aβ. Cells take up soluble Aβ via macropinocytosis and the LDL receptor, protein‐mediated pathway.^[^ ^14^, ^37^, ^38^ ^]^ In contrast, fibrillar forms of Aβ interact with innate immune receptor complexes on the surface of microglia, initiating intracellular signaling cascades that stimulate phagocytosis.^[^ ^32^, ^39^ ^]^

In human brain tissue, high levels of soluble Aβ have been shown to be correlated with increased AD severity.^[^ ^40^, ^41^ ^]^ Based on our investigation, it seems possible that Lyzl4 is not directly involved in microglial phagocytosis. However, increased levels of Lyzl4 in microglia resulted in enhanced Aβ degradation when co‐cultured with pHrodo‐Aβ, indicating that Lyzl4 may promote the microglial clearance of Aβ following phagocytosis. However, following RNA knockdown of Lyzl4, we did not observe any significant differences in Aβ clearance. We propose that other Aβ‐clearance pathways, such as those mediated by proteases or phagocytosis‐enhancing receptors, may be upregulated in response to Lyzl4 knockdown, potentially compensating for its loss of function. To further elucidate this, additional experiments are required to assess the expression and activity of these functionally redundant immune pathways. Such investigations could employ pathway‐specific inhibitors or genetic approaches to evaluate shifts in these signaling pathways following Lyzl4 knockdown.

Our results also suggest that Lyzl4 overexpression facilitates microglial engulfment of soluble Aβ by fluid‐phase micropinocytosis (Video S5, Supporting Information). In vitro, Aβ and Lyzl4 co‐localize mainly with Rab7 (late endosomes) and Lamp1 (lysosomes), but not with EEA1 (early endosomes). Macropinocytic vesicles are formed by the closure of membrane ruffles; once Aβ is internalized, it appears to be transported to late endosomes and lysosomes via the direct fusion of the macropinocytic vesicle to late endocytic vesicles. Previous studies have suggested that microglia eliminate soluble Aβ via fluid‐phase macropinocytosis,^[^ ^14^ ^]^ and our results indicate that cytochalasin D impairs the ability of microglia to uptake soluble Aβ. Cytochalasin D inhibits actin polymerization, which is essential for forming membrane ruffles and subsequent macropinosomes during fluid‐phase macropinocytosis.^[^ ^42^ ^]^

In addition, our WGCNA results position *Lyzl4* as a key gene within the module most strongly associated with AD‐related changes in microglia. Further network analysis within this module demonstrates the specific connections of Lyzl4 with other genes. GO enrichment analysis of these Lyzl4‐related genes reveals significant pathways related to response to external stimuli, stress response, defense response, and other critical processes (Figure S1E, Supporting Information), underscoring the potential involvement of Lyzl4 in AD‐related mechanisms. Moreover, our transcriptomic analysis also reveals increased expression of several inflammatory and disease‐associated genes, including Srgn, Trem2, and Tyrobp, in 12‐month‐old APP/PS1 versus WT mice. Conversely, anti‐inflammatory genes such as Cx3cr1 and Tmem119 are downregulated in microglia from 12‐month‐old APP/PS1 mice (Figure S1C, Supporting Information). This differential gene expression profile supports the role of microglia activation in AD pathology, indicating that by 12 months of age, microglia have transitioned to an activated state.

Following stereotactic injection, the Lyzl4‐AAV‐injected hemisphere from 5‐month‐old APP/PS1 mice contained fewer Aβ plaques compared to the control AAV‐injected hemisphere. We hypothesize that the incomplete clearance of Aβ following Lyzl4 overexpression in vivo could be attributed to three factors: still‐insufficient amounts of Lyzl4, inadequate waiting time for experimental data collection, or the potential involvement of other cell types in Aβ clearance that are unaffected by Lyzl4 expression. Additionally, relying solely on the assessment of Aβ burden is not a comprehensive enough tool to assess the impact on AD prognosis. Future work must add behavioral studies, such as water maze and minefield experiments, to evaluate whether Lyzl4 overexpression also drives an improvement in cognitive performance. Further investigation is also required to elucidate the precise molecular mechanisms by which Aβ stimulates elevated Lyzl4 expression in microglia and how enhanced Lyzl4 levels facilitate Aβ degradation. However overall, our results demonstrate that microglial Lyzl4 plays an important role in Aβ degradation. Augmenting Lyzl4 expression and function in the brain could present an effective strategy for facilitating Aβ clearance and countering amyloid pathology in AD.

## 4 Experimental Section

### Mice

All mice were maintained as previously reported.^[^ ^43^ ^]^ APPswe/PS1ΔE9 double‐transgenic mice used in this study were obtained from the Model Animal Research Center of Nanjing University (Nanjing, China), and were originally bred from B6.Cg‐Tg(APPswe/PS1ΔE9)85Dbo/Mmjax mice (JAX#03 4832) obtained from The Jackson Laboratory. C57BL/6 J WT littermates served as controls. Mice were housed in IVC cages under Specific Pathogen‐Free (SPF) conditions, with a temperature of 23 °C and a humidity range of 50% to 60%, and with circadian rhythm illumination. Pups aged 21 to 28 days were separated from their parental cages and genotyped using ear‐biopsy samples. The DNA extracted from the biopsy samples was PCR‐amplified using primers specific for APP and PS1 sequences. The Animal Use and Care Committee of Shenzhen Peking University – The Hong Kong University of Science and Technology Medical Center (SPHMC) approved all procedures (protocol number 2022‐014), with efforts undertaken to minimize the number of animals used and to reduce suffering.

### Primary Microglia Isolation by Magnetic‐Activated Cell Sorting (MACS) or Fluorescence‐Activated Cell Sorting (FACS)

Primary microglia isolation was performed as previously reported.^[^ ^44^ ^]^ Neonatal microglia were isolated by using MACS from p7 C57BL/6 J mice. Cerebral tissue was isolated, meninges removed and the brains of three mice pooled in a C‐tube for tissue dissociation with the Neural Tissue Dissociation Kit (130‐092‐628, Miltenyi Biotec) on program 37C_NTDK_1 of the gentleMACS Octo Dissociator with Heaters (130‐096‐427, Miltenyi Biotec) allowing for simultaneous enzymatic and mechanical tissue disruption.

Adult microglia were isolated from WT/ AD mice at 2, 4, 6, 9, or 12 months of age old (five groups). Mice were transcardially perfused under deep anesthesia with 1 × PBS, then the brain was removed, dissected, and rinsed in Hanks’ Balanced Salt solution without calcium and magnesium (HBSS, 14 170 138, Thermo Fisher). Next, after removing the meninges, the brain was cut into small pieces using a sterile scalpel, and the samples were centrifuged at 300 × g for 2 min at room temperature before the supernatant was aspirated carefully. Samples from a single brain were pooled as a single experimental group.

Enzymatic cell dissociation was performed using an Adult Brain Dissociation Kit (130‐107‐677, Miltenyi Biotec) on program 37C_ABDK_01 of the gentleMACS Octo Dissociator with Heaters. For MACS, the cell suspension was washed with HBSS and the microglia were magnetically labeled with CD11b microbeads (130‐097‐678, Miltenyi Biotec), according to the manufacturer's protocol. The resulting cell were passed through LS columns (130‐042‐401, Miltenyi Biotec) and placed on an OctoMACS manual separator to collect the CD11b ‐positive cell fraction. For FACS, the cell suspension was resuspended in FC receptor blocking solution (553 141, BD Biosciences) and incubated on ice for 10 min. Following incubation, the cells were co‐stained on ice in the dark for 30 min with PE‐Cy7‐labeled CD45 (103 114, BioLegend) and PE‐labeled CD11b (101 208, BioLegend). After staining, the cells were washed with PBS, centrifuged, and resuspended in FACS buffer (1% FBS, 2 mm EDTA, 25 mm HEPES, and 1:500 RNA inhibitor in PBS). The cells were then incubated with 7AAD (420 403, BioLegend) for 10 min prior to sorting. Data acquisition and analysis were performed using BD FACS Diva v8.0.1 software.

### Cell Culture

BV2 mouse microglial cells were cultured in DMEM (GIBCO) containing 10% fetal bovine serum (GIBCO), 50 U ml^−1^ penicillin, and 50 µg ml^−1^ streptomycin (GIBCO). For primary microglia, cell culture plates (Corning, 35 mm dish) were prepared one day prior to microglia isolation. First, wells were coated with 20 µg ml^−1^ poly‐L‐lysine (PLL) (Sigma, 2636‐25MG) in PBS overnight at 37 °C, 5% CO2. The next day, wells were washed twice with PBS. CD11b labeled cells were flushed from the MS column and cultured in DMEM containing 10% fetal bovine serum, 50 U ml^−1^ penicillin, 50 µg ml^−1^ streptomycin and 0.25% L‐glutamine (Invitrogen). All cells were maintained in a humidified incubator containing 5% CO_2_ at 37 °C.

### RNA Extraction and Real‐Time PCR Analysis

RNA extraction and real‐time PCR analysis were performed as previously reported.^[^ ^25^ ^]^ Microglia were isolated by MACS and RNA was extracted using an RNeasy Micro Kit (74 004, Qiagen) according to the manufacturer's instructions, which included a step involving incubation with DNase. Purified RNA was quantified using a NanoDrop 2000 (Thermo Scientific) and Agilent Technologies Bioanalyzer 2100 RNA Pico chips (5067‐1513, Agilent Technologies), according to manufacturer instructions; the RNA integrity number (RIN) in all cases was >9. Quantitative RT‐PCR was performed in triplicate in 96‐well plates by using a qPCR machine (LC480, Roche) and SYBR Green I Master mixture (4 887 352 001, Roche) for the detection of amplification products. The following thermocycling protocol was used: initial denaturation at 95 °C for 10 min, followed by 40 amplification cycles of 95 °C for 15 s and 60 °C for 1 min, and a final cycle at 25 °C for 15 s. The relative quantification of mRNA expression was performed using the comparative cycle method to obtain the following ratio: gene of interest/Gapdh. The relative quantification of gene expression levels was performed using the 2^−ΔΔCt^ method. All primers were designed using NCBI Primer‐BLAST; primers were designed to be ≈200‐bp long. All primers are listed in Table S1 (Supporting Information).

### RNAscope and Immunohistochemistry

RNAscope and immunohistochemistry were performed as previously reported.^[^ ^25^, ^43^ ^]^ Mice were deeply anesthetized using pentobarbital, transcardially perfused with ice‐cold PBS until the irrigation fluid was completely clear, then perfused with ice‐cold 4% paraformaldehyde (PFA) for 10 min. Brains were removed, fixed in 4% PFA in 4 °C refrigerator for 12 h, dehydrated using an ethanol dilution series, embedded in molds containing Tissue‐Tek OCT, and frozen in dry ice. The OCT‐embedded brain samples were cut into 16 µm coronary sections and placed onto Fisherbrand Superfrost Plus microscope slides (Thermo Fisher Scientific; 12‐550‐15). RNAscope experiments were performed using a Manual Fluorescent Multiplex kit v2 (323 100, ACDbio), following the manufacturer's recommendations. Briefly, slices were incubated with hydrogen peroxide, then target retrieval was performed in a boiling bath beaker. Next, protease digestion was performed for 20 min at room temperature using Protease III for fixed frozen tissues, as provided in the kit. Afterward, probe hybridization was conducted for 2 h at 40 °C. A dual probe set containing Mm‐Itgam‐c3 (311 491) and MmLyzl4‐c1 (582 031) served as the common probe in each set. Nuclei were visualized using 4′,6‐Diamidino‐2‐phenylindole (DAPI).

For immunohistochemistry, after blocking and permeabilization for 1 h in PBS containing 4% bovine serum albumin (BSA) and 0.4% Triton X‐100, the slices were incubated at 4 °C overnight with primary antibodies diluted in PBS. Then, slides were washed with PBS and incubated with secondary antibodies in PBS at room temperature for 2 h. Nuclei were visualized using DAPI. Images were captured as z‐stacks using a Zeiss LSM980 confocal microscope (Carl Zeiss) or a Zeiss Celldiscoverer 7 (Carl Zeiss) to generate maximum‐intensity. Lastly, blinded counting was used to quantify single‐positive and double‐positive cells. All parameters remained constant between images to allow unbiased detection. The following antibodies were used for staining: anti‐IBA1 (019‐19741, Wako); Purified anti‐β‐Amyloid (6E10, Biolegend); anti‐EEA1(610 457, Pharmingen); anti‐Rab7(9367T, CST); anti‐Lamp1(ab24170, Abcam); donkey anti‐rabbit Alexa Fluor 488 (A11001, Invitrogen; 1:500); and goat anti‐mouse Alexa Fluor 555 (A21422, Invitrogen; 1:500).

### Sequence Analysis

A homology search was conducted against the GenBank database (http://www.ncbi.nlm.nih.gov/BLAST/) using the BLAST algorithm. Multiple sequence alignments were performed using ClustalW programs (http://www.ebi.ac.uk/clustalw/) with default settings.

### Plasmids and Viral Vectors

BV2 cells or primary microglia were transfected with CMV‐Lyzl4‐EGFP plasmids. The Lyzl4 overexpression vector was constructed by homologous recombination using the pLVX‐IRES‐tdTomato plasmid for primary microglia. For cell transfection assays, cells were first seeded on cell culture plates and grown to a confluence of ≈50%. Next, cells were transfected with Lyzl4 or the vector using ViaFect Transfection Reagent (Promega, United States) according to the manufacturer's protocol and cultured at 37 °C with 5% CO2 for 48–72 h. For stereotactic injection, the pAAV‐Iba1p‐Lyzl4‐3FLAG‐P2A‐EGFP‐SV40 PolyA plasmid was assembled by homologous recombination of an AAV backbone linearized from the modified CV287 plasmid by PCR.

### Aβ Preparation and Cell Treatment

Lyophilized Aβ_1–42_ (Sigma) was dissolved in 1.0% NH_4_OH, then diluted with 1×PBS to a concentration of 1 mg mL^−1^. Before treatment, the Aβ reaction mixture was oligomerized at 37 °C overnight. BV2 and primary microglia were treated with Aβ at concentrations of 1, 2, and 4 µm, respectively. Samples were collected at 12, 24, 60, and 100 h after treatment. Lyzl4 expression was assessed by qPCR.

### Transfection of siRNA

For the siRNA transfection of primary microglia, 50 nm siRNA (RiboBio) was added to the cells using RNA iMAX Reagent (Sigma) according to the manufacturer's guidelines. After 24 h of transfection, the cells were treated with oligomerized Aβ and harvested at 0, 8, 16, and 24 h post‐treatment. The siRNAs used in this study can be found in Table S2 (Supporting Information).

### Preparation of Fluorescent Phagocytic Material

The 3 um Polybead‐Amino Microspheres (Polyscience) were rinsed with PBS twice by centrifugation (13,000 g, 5 min, room temperature) before further labeling. Each prey particle was labeled with pHrodo iFL Green STP Ester (Thermo Fisher) by incubation at room temperature in the dark for 45 min in PBS with 0.1 m sodium bicarbonate at the following ratios: 2.5% (w/v) beads with 10 µm pHrodo;^[^ ^45^ ^]^ 1:10 (molar ratio) for oligomerized Aβ (at a concentration of 1 mg mL^−1^) to pHrodo. Dye concentrations were optimized for the maximum level of labeling that did not produce a fluorescent signal when particles alone were imaged at a neutral pH. pHrodo‐Polybead‐Amino Microspheres were centrifuged at 13,000 g for 5 min at room temperature and washed a total of four times after labeling to remove excess dye before storage at 4 °C. pHrodo‐Aβ oligomers were added to Micro Bio‐Spin P‐6 Gel Columns for affinity chromatography to remove excess pHrodo.

### Live‐Cell Imaging and Immunofluorescent Microscopy

BV2 cells or primary microglia were transfected with CMV‐Lyzl4‐EGFP plasmids or the empty vector on cell culture plates. A Zeiss Celldiscoverer 7 was used to image all cells (Carl Zeiss), with a heated stage and objectives to maintain the temperature of the culture media at 37 °C. LysoTracker (L12492, Invitrogen) was performed according to the manufacturer's instructions. In brief, the 1 mm probe stock solution was diluted to the 50–75 nm final working concentration. When microglia reached the desired confluence, the medium was removed from the dish and replaced with the prewarmed (37 °C) probe‐containing medium. Cells were then incubated for 30 min to 2 h under growth conditions.

### In Vitro Phagocytosis Assay

Primary microglia were transfected with Lyzl4 or the empty vector on cell culture plates. After 24 h, the cells were collected, counted, and re‐seeded at appropriate ratios. The following day, the supernatant was removed, and pHrodo‐Polybead‐Amino Microspheres (at a ratio of 10:1 with the cells) and pHrodo‐Aβ oligomers (5 ug mL^−1^) were diluted in serum‐free medium added to the culture plates.^[^ ^46^ ^]^ For each time point, in order to combine data from independent experiments, the percentage of microglia was quantified that engulfed beads or Aβ oligomers out of the total microglial population per field of view. The experiment was divided into three groups: lentiviral infection group, negative inhibitor group (Cytochalasin D), and blank control group. Cytochalasin D (Merck) was administered to the cells at a concentration of 10 µm and added to the cells 5 min prior to the introduction of the phagocytic material. For imaging microglia, a 50× objective was used on Zeiss Celldiscoverer 7. A heated stage and objectives were used to maintain the temperature of the culture media at 37 °C. After continuous monitoring for 24 h, the experimental data were exported and analyzed using ImageJ software.

### ELISA

Brain tissue or cells were lysed in RIPA buffer (P0013K, Beyotime) with 1 mm PMSF (ST506, Beyotime) and a protease inhibitor cocktail (5871S, CST). Levels of Aβ_1‐42_ were analyzed by using the Quantikine ELISA Human Amyloid β (aa1‐42) Immunoassay (DAB142). Standard sandwich ELISA measurements of soluble Aβ42 were performed based on the manufacturer's instructions. Briefly, 96‐well plates were washed twice with Wash Buffer and prepared with necessary reagents, working standards, and samples. Excess microplate strips were removed and stored in a sealed foil pouch with a desiccant pack. After adding 100 µL of standard, control, or sample per well, the plates were incubated for 2 h at 2–8 °C. Following the incubation, wells were aspirated and washed four times, ensuring complete liquid removal. Next, 200 µL of cold Human Amyloid β (aa1‐42) Conjugate was added to each well and incubated for an additional 2 h at 2–8 °C. Subsequently, wells were aspirated and rewashed, followed by adding 200 µL of Substrate Solution and incubating for 30 min at room temperature, protected from light. After adding 50 µL of Stop Solution, the color change in wells was monitored, and plates were gently tapped for thorough mixing if necessary. The optical density of each well was determined within 30 min using a microplate reader set to 450 nm with wavelength corrections applied at 540 or 570 nm.

### Stereotactic Injection

Stereotactic injection was performed as previously reported.^[^ ^47^ ^]^ After anesthetization with pentobarbital, mice were placed in a stereotactic frame (RWD, China) for intraperitoneal injection. A midline incision was made on the scalp, and one hole was drilled in the skull according to the following coordinates: anteroposterior (AP) 1.5 mm, lateral (LAT) 3.0 mm, dorsoventral (DV) 1.9 mm (bregma as reference). 5E + 9 vg pAAV‐Iba1p‐Lyzl4‐3FLAG‐P2A‐EGFP‐SV40 PolyA‐overexpressing adenovirus or control virus was injected at a rate of 0.2 µl min^−1^ with 26 G needles in a 5 µl Hamilton syringe with a compact infusion pump (2 µL injection volume). After the injection, the needle was maintained for another 10 min, then raised at a rate of 1 mm per 5 min. The bone hole was sealed using bone wax, and mice were placed on a 37 °C heating plate until awake and returned to home cages. Mice were subjected to cardiac perfusion, and frozen sections were taken 4 weeks after injection.

### Cytokine Level Detection Using Milliplex Kits

Cell lysis buffer (9803, CST) was diluted with double‐distilled water, and a 1 mm protease inhibitor cocktail (set III, EDTA‐Free; 539 134, Millipore) was added before use. After isolating primary microglia via FACS, microglia were treated with cell lysis buffer, homogenized, and centrifuged for 10 min at 14000 × *g* at 4 °C to eliminate precipitates. Duplicate supernatant samples were collected to measure total protein concentrations using the Bradford protein assay. Cytokine levels in each sample were assessed using the MCYTOMAG‐70K Mouse Cytokine magnetic kit (EMD Millipore), with data analyzed through Milliplex Analyst 5.1 software (EMD Millipore). Noise correction involved subtracting the mean of two technical control wells for each secreted soluble factor.

### Dataset Acquisition and Analysis

The dataset includes previously‐generated microglia samples from both WT and APP/PS1 model mice at 2, 4, 6, 9, and 12 months of age.^[^ ^25^ ^]^ This previous study was described in detail and accessible via the GEO series accession number GSE137028.

Briefly, paired‐end reads were aligned using Hisat2 v2.0.53.^[^ ^48^ ^]^ Feature Counts v1.5.0‐p34^[^ ^49^ ^]^ were employed to enumerate the reads aligned to each gene. Subsequently, the expression level of each gene was quantified as fragments per kilobase of transcript per million mapped reads (FPKM). Differential expression analysis was performed using DESeq2^[^ ^50^ ^]^ in R, with genes having an adjusted p‐value < 0.05 and log2 fold change >0.5 considered significant. The WGCNA^[^ ^26^ ^]^ package in R was employed to construct a weighted gene co‐expression network (WGCNA). Prior to analysis, a log2(n+1) transformation was applied to the gene expression data. The median absolute deviation (MAD) was then calculated for each gene and selected the top 2500 genes based on their MAD values to focus on the most variable genes. A soft‐thresholding power of 6 was determined, ensuring the network achieved a scale‐free topology (Scale Free Topology Model Fit, signed *R^2^* > 0.8). The network of genes connected to Lyzl4 was visualized, highlighting the top 25% of genes most correlated with Lyzl4. GO‐BP^[^ ^51^ ^]^ enrichment analysis of these genes was performed to identify significant biological processes.

Additional publicly available datasets were used throughout the study for validation or comparison.

First: RNA‐seq transcriptomics data of rTg4510 tau transgenic mouse models were obtained from GEO (accession number GSE123467).^[^ ^28^ ^]^ This dataset compares microglial cell gene expression changes in rTg4510 tau transgenic mice and wild‐type mice at four age groups (2, 4, 6‐, and 8 months). As the dataset provides log2‐transformed and median‐normalized data, the log‐transformed data was converted back to the original scale using the exponential function and subsequently compared Lyzl4 expression levels.
Second: Single‐nucleus RNA sequencing (snRNA‐seq) data of mouse cerebral aging within the frontal cortex and striatum were obtained from GEO (accession number GSE207848).^[^ ^30^ ^]^ The R package Seurat^[^ ^52^ ^]^ was utilized for data analysis and visualization. Lyzl4 gene expression across various human tissues was analyzed using data from the Tissue‐specific Gene Expression and Regulation Database (TissGDB).^[^ ^53^ ^]^
Finally: RNA‐seq transcriptomics data of APP^SAA^KI/KI mouse models were obtained from GEO (accession number GSE158152).^[^ ^27^ ^]^

### Statistical Analysis

Statistical analyses were performed using the GraphPad Prism 8.00 software (GraphPad Software, La Jolla, CA, USA) or as indicated otherwise for RNAseq data. Most data were analyzed using one‐way ANOVA followed by Dunnett post hoc test for comparisons involving three or more samples, and two‐sample unpaired t‐tests were used for comparing 2 samples; *p* < 0.05 was considered statistically significant.

## Conflict of Interest

The authors declare no conflict of interest.

## Author Contributions

J.P. and J.Z. contributed equally to this work. J.P. designed the study, performed experiments, analyzed data, and wrote the manuscript. J.Z. performed experiments, analyzed data, and wrote the manuscript. J.G. and S.S.H. performed experiments and analyzed data. J.O. analyzed data and wrote the manuscript. J.W. conceived and supervised the study, performed experiments, wrote the manuscript, and provided funding.

## Supporting information



Supporting Information

Supplemental Video 1

Supplemental Video 2

Supplemental Video 3

Supplemental Video 4

Supplemental Video 5

## Data Availability

The data that support the findings of this study are available in the supplementary material of this article.
