# Supplementary material for: Microglial Lyzl4 Facilitates β‐Amyloid Clearance in Alzheimer's Disease
Source: Adv Sci (Weinh). 2024 Nov 18;12(2):2412184. doi: 10.1002/advs.202412184 (PMC11727385; doi:10.1002/advs.202412184)
Supplement: Supplementary file 1 — Supporting Information [file ADVS-12-2412184-s005.docx]

**Supplementary Information:**

**Table S1: Primer list.**

| **Primers** |  |  |
| --- | --- | --- |
| **Lyzl1** | Forward | GCATAGTCGCAGAATCCA |
|  | Reverse | CAGCCCTGCCAATAGTTC |
| **Lyzl3** | Forward | GGCCAAGGTCTTCAGTCG |
|  | Reverse | ACAGCCATCCACCCAGTC |
| **Lyzl4** | Forward | ATGCAGCTGTACCTGGTGCTTCT |
|  | Reverse | GCTGGTTTATTCTGCACCTTGTACC |
| **Lyzl6** | Forward | ATCCATCGCTGTAGTTTG |
|  | Reverse | TGAAATGAGGTTGGGACT |
| **GAPDH** | Forward | AACTTTGGCATTGTGGAAGG |
|  | Reverse | GGATGCAGGGATGATGTTCT |

**Table S2: si-RNA probes.**

| **Name** | **Probe sequence（5’-3’）** |
| --- | --- |
| **si-Lyzl4-1** | GGCTAAGATGCTCTATGAT |
| **si-Lyzl4-2** | TCAGATTCGTGACAATGAA |

**
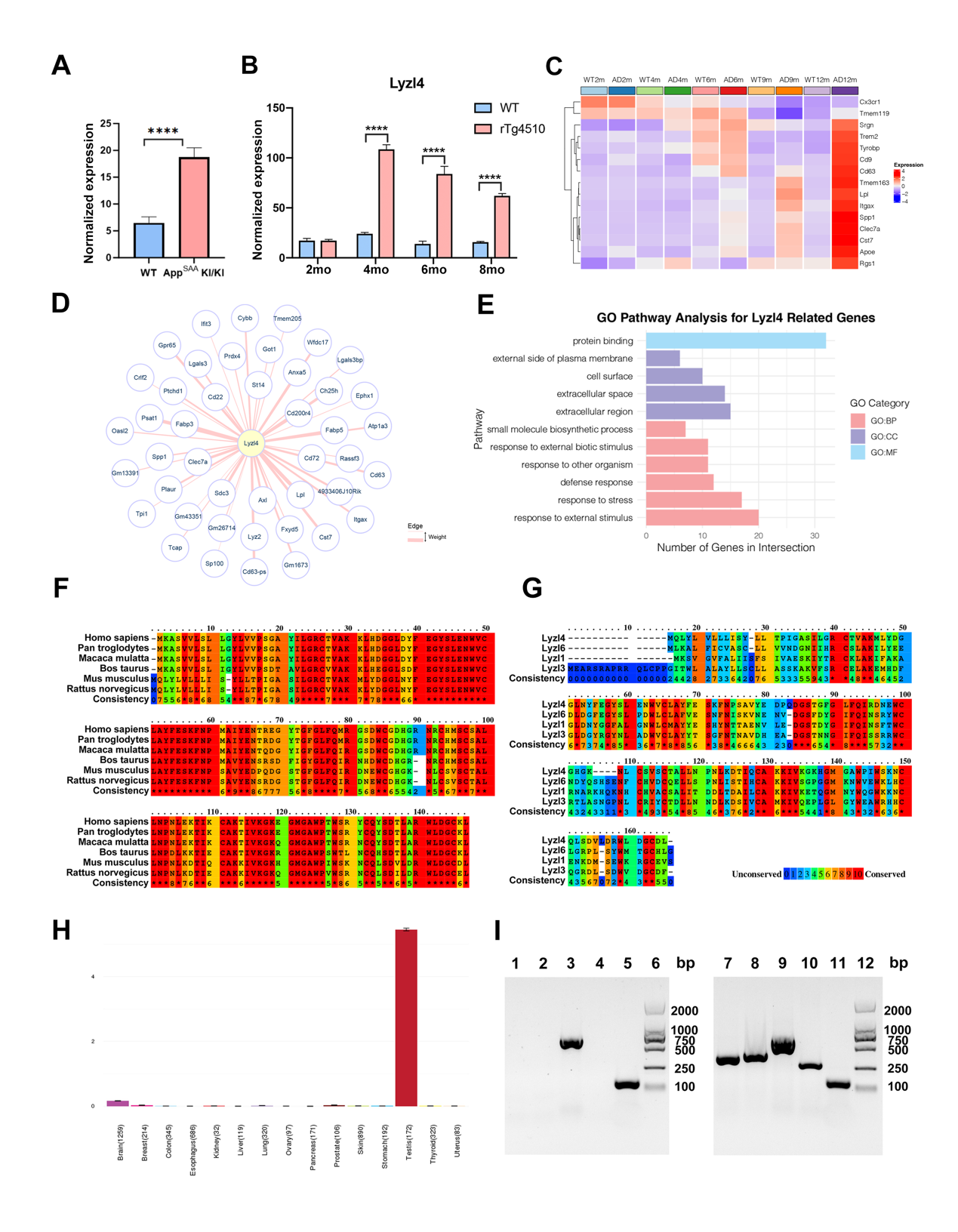
**

**Figure S1:** Lyzl4 gene expression in AD models, tissue distribution, c-type lysozyme family homology, and conservation across species.

(A) Normalized expression levels of Lyzl4 in the APP^SAA^KI/KI mouse model of AD and control (WT) mice at 8 months of age. Unpaired t-test; data are shown as means ± SEM; ****p < 0.0001.

(B) Normalized expression levels of Lyzl4 in the rTg4510 TAU mouse model of AD and control (WT) mice at 2, 4, 6, and 8 months of age. Unpaired t-test; data are shown as means ± SEM; ****p < 0.0001.

(C) Heatmap displaying the expression levels of inflammatory and disease-associated genes in astrocytes from wild-type (WT) and AD mice at 2, 4, 6, 9, and 12 months of age. Each row represents a gene, and each column represents a sample group (WT or AD at different ages). The color scale indicates the level of gene expression, with red representing upregulation and blue representing downregulation.

(D) Network diagram illustrating the relationship between the gene *Lyzl4* (from node) and other genes within module 3, identified by WGCNA. Highlighting the top 25% of genes most correlated with *Lyzl4*. Each circle denotes a gene, with thicker edges indicating stronger gene co-expression relationships.

(E) GO enrichment analysis for genes related to Lyzl4 (identified in panel D). Pathways with adjusted p-values < 0.05 are displayed.

(F) Conservation analysis of the Lyzl4 sequence across different species.

(G) Homologous sequence alignment of Lyzl1, Lyzl3, Lyzl4, and Lyzl6.

(H) Lyzl4 gene expression across human tissues (TissGDB).

(I) RT-PCR analysis showing the expression of c-type lysozyme-like family members in brain and testis tissues. Lanes 1–6: Brain tissue expression; Lanes 7–12: Testis tissue expression. Specific lanes represent: 1. Lyzl1 (346bp), 2. Lyzl3 (382bp), 3. Lyzl4 (602bp), 4. Lyzl6 (261bp), 5. GAPDH (132bp, control), and 6. Ladder markers. Testis tissue: 7. Lyzl1 (346bp), 8. Lyzl3 (382bp), 9. Lyzl4 (602bp), 10. Lyzl6 (261bp), 11. GAPDH (132bp), and 12. Ladder markers.

**
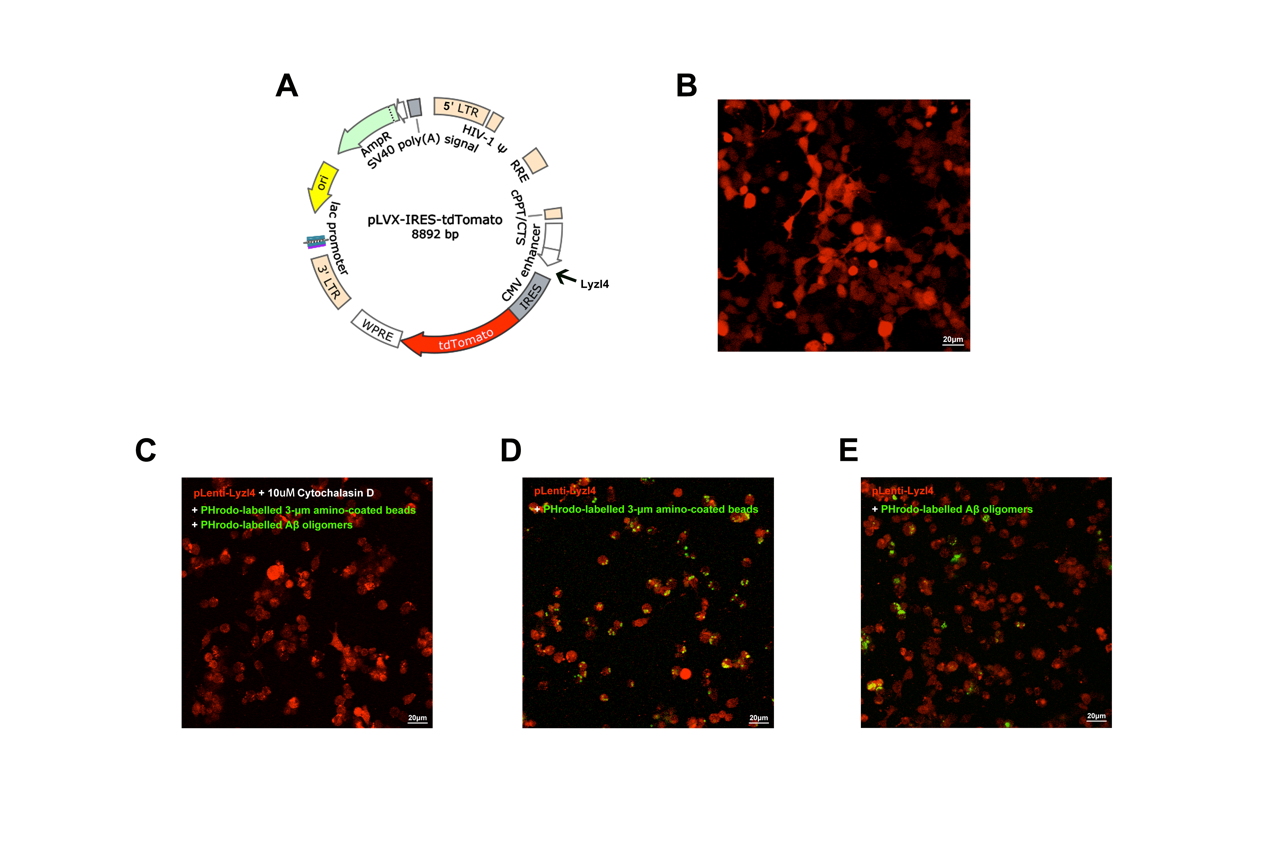
 Figure S2:** Lentiviral-mediated overexpression of Lyzl4 in microglia enhances clearance of Aβ.

(A) Construction of lentiviral vector for overexpression of Lyzl4.

(B) Representative epifluorescence image of microglia infected with lentiviral vectors expressing Lyzl4 (555 nm excitation, red). Scale bar: 20 μm.

(C) Representative epifluorescence images of the phagocytosis of pHrodo-labelled 3-µm amino-coated microspheres and pHrodo-Aβ oligomers (green) by Lyzl4-overexpressing microglia (red). Microglia were treated with actin-polymerization inhibitor cytochalasin D to inhibit phagocytic behavior. Scale bar: 20 μm.

(D) Representative epifluorescence images of the phagocytosis of pHrodo-labelled 3-µm amino-coated microspheres (green) by Lyzl4-overexpressing microglia (red). Scale bar: 20 μm.

(E) Representative epifluorescence images of the phagocytosis of pHrodo-Aβ oligomers (green) by Lyzl4-overexpressing microglia (red). Scale bar: 20 μm.

**Video S3:** Time-lapse video of the phagocytosis of pH-sensitive fluorescent beads by BV2 cells.

**Video S4:** Time-lapse video of the phagocytosis of pH-sensitive fluorescent Aβ oligomers by BV2 cells.

**Video S5:** Time-lapse video of the phagocytosis of pH-sensitive fluorescent beads by primary microglia.

**Video S6:** Time-lapse video of the phagocytosis of pH-sensitive fluorescent Aβ oligomers by primary microglia.

**Video S7:** Time-lapse video of the phagocytosis of pH-sensitive fluorescent soluble Aβ by primary microglia.
